# Characterizing Polymer Hydration Shell Compressibilities with the Small-System Method

**DOI:** 10.3390/nano10081460

**Published:** 2020-07-25

**Authors:** Madhusmita Tripathy, Swaminath Bharadwaj, Shadrack Jabes B., Nico F. A. van der Vegt

**Affiliations:** Eduard-Zintl-Institut für Anorganische und Physikalische Chemie, Technische Universität Darmstadt, 64287 Darmstadt, Germany; tripathy@cpc.tu-darmstadt.de (M.T.); bharadwaj@cpc.tu-darmstadt.de (S.B.); barnabas@cpc.tu-darmstadt.de (S.J.B.)

**Keywords:** small system method, thermodynamics of small systems, hydration shell thermodynamics, finite size correction

## Abstract

The small-system method (SSM) exploits the unique feature of finite-sized open systems, whose thermodynamic quantities scale with the inverse system size. This scaling enables the calculation of properties in the thermodynamic limit of macroscopic systems based on computer simulations of finite-sized systems. We herein extend the SSM to characterize the hydration shell compressibility of a generic hydrophobic polymer in water. By systematically increasing the strength of polymer-water repulsion, we find that the excess inverse thermodynamic correction factor ($\Delta 1/\Gamma_{s}^{\infty }$) and compressibility ($\Delta \chi_{s}$) of the first hydration shell change sign from negative to positive. This occurs with a concurrent decrease in water hydrogen bonding and local tetrahedral order of the hydration shell water. The crossover lengthscale corresponds to an effective polymer bead diameter of 0.7 nm and is consistent with previous works on hydration of small and large hydrophobic solutes. The crossover lengthscale in polymer hydration shell compressibility, herein identified with the SSM approach, relates to hydrophobic interactions and macromolecular conformational equilibria in aqueous solution. The SSM approach may further be applied to study thermodynamic properties of polymer solvation shells in mixed solvents.

## 1. Introduction

Although the laws of statistical mechanics are customarily used in computer simulations to relate thermodynamical properties of a broad range of systems to interactions between their constituent components, there remains a caveat: computer simulations are performed on finite-sized systems in which the thermodynamic properties may deviate from the corresponding properties in the thermodynamic limit (TL) of large systems [1,2]. An example is the incorrect asymptotic behavior of the radial distribution function (RDF), g(r), obtained by computer simulation of a closed system with a finite number of particles. Routes for calculating thermodynamic properties from integrals that involve the RDF are sensitive to subtle changes in the asymptotic tail behavior of the RDF and, therefore, show size dependencies in practical calculations. This issue is well known [3,4,5] and has received renewed attention in recent years, in particular, in applications of the Kirkwood-Buff theory of solutions and the calculation of Kirkwood-Buff integrals (KBI) [4,5,6,7,8,9].

The small-system method (SSM) by Schnell et al. [10,11] provides another route for computing thermodynamic properties in the TL from small scale fluctuations. This method uses a small-system scaling relation, derived based on the thermodynamics of small systems by Hill [12,13], to obtain thermodynamic properties corresponding to the TL. For example, the scaling relation for the thermodynamic correction factor, Γ, of a single-component small system is given by [10]

(1) $$1/\Gamma \left(L\right)=1/\Gamma^{\infty }+\frac{c}{L}$$

where *L* is the linear dimension of a small system with volume $V=L^{3}$, *c* is a constant that does not depend on *L*, and $\Gamma^{\infty }$ is the thermodynamic correction factor in the TL where V→∞, 〈N〉→∞, and the particle number density ρ=〈N〉/V is constant. $\Gamma^{\infty }$ is related to the isothermal compressibility ($\chi_{T}$) according to

(2) $$1/\Gamma^{\infty }=\rho k_{B}T\chi_{T}.$$

The inverse thermodynamic correction factor in Equation (1) follows from density fluctuations in the grand-canonical ensemble

(3) $$1/\Gamma \left(L\right)=\frac{\langle N^{2}\rangle -{\langle N\rangle }^{2}}{\langle N\rangle },$$

where *N* is the number of particles and 〈·〉 indicates ensemble averages. In practice, 1/Γ(L) is calculated for small open systems of varying linear dimension *L* embedded in a large particle reservoir (closed simulation box of volume $V_{b}>>L^{3}$). The scaling relation (Equation (1)) is then observed for small-system sizes that obey $V_{b}^{1/3}>L>\xi$ (where ξ is the correlation length). The SSM has been successfully applied to pure component systems as well as binary and multicomponent systems [5,11,14,15,16]. Very recently, the method has been further employed to study the thermodynamic properties of confined fluids in nanopores [17,18]. In a spirit similar to the SSM, Jamali et al. [19] exploited the scaling of the elements of the diffusivity matrix, which depends on Γ, with system size to estimate their TL value for ternary molecular and model mixtures.

Recently, Trinh et al. [20,21] have extended the SSM to study the thermodynamic properties of adsorbed CO${}_{2}$ and CH${}_{4}$ layers on graphite and activated carbon surfaces. Motivated by these studies, we herein extend the SSM to investigate the compressibility of polymer hydration shells. The TL (1/L→0) of an extended hydration shell will be considered for a fully stretched linear chain with *L* defined along the direction of the chain. The hydration shell compressibility of hydrophobic polymers relates to hydrophobic interactions and pressure dependencies of polymer (coil-to-globule) collapse equibria in water. We are, in particular, interested in the role of a crossover length scale in hydrophobic hydration of macromolecules. While hydration thermodynamics of small hydrophobic solutes is governed by density fluctuations in pure water, hydration of large hydrophobic solutes is governed by the (de)wetting of their surfaces [22,23]. The crossover length scale, above which a macroscopic thermodynamic description applies, has been investigated based on computer simulations that studied the hydration thermodynamics of small and large hydrophobic solutes [23,24,25,26]. Susceptibilities of extended hydration shells to external perturbations have been characterized more recently [27,28] and further demonstrates the role of dewetting of hydrophobic surfaces in the thermodynamics of biomolecular interactions such as protein-protein and ligand-protein interactions.

We will herein ask if a small-to-large crossover can be observed in the polymer hydration shell compressibility by systematically increasing the range of the repulsive interaction between beads of a generic polymer and water. Our results demonstrate that $\Delta 1/\Gamma_{s}^{\infty }$ and $\Delta \chi_{s}$, i.e., the inverse thermodynamic correction factor and the compressibility of the first hydration shell in excess to the corresponding properties of a shell of exactly the same radial and lateral dimensions in pure water, exhibit a crossover from negative to positive values at intermediate strengths of the polymer bead-water repulsion. The observed crossover corresponds to an effective polymer bead Lennard-Jones diameter of approximately 0.7 nm. This crossover length scale in hydrophobic hydration agrees with earlier work on hydrophobic hydration of spherical solutes in which a relation to the Egelstaff–Widom lengthscale, i.e., the product of the surface tension and compressibility, of water was demonstrated [29]. In the present work, we observe this small-to-large crossover for all lateral dimensions *L* of the small system (first hydration shell). However, $\Delta 1/\Gamma_{s}\left(L\right)$ is independent of *L* for effective polymer bead sizes below 0.7 nm, while it increases with *L* for hydrophobic polymers with larger effective bead sizes. This indicates that only for effective polymer bead sizes larger than 0.7 nm, the overall exposed hydrophobic polymer surface area impacts the excess thermodynamic properties of hydrophobic polymer hydration shells and causes a dependence of these properties on the linear polymer dimension whose limiting values can be determined with the small system method.

The remainder of this paper is structured as follows. In Section 2, we discuss the details of the simulations. In Section 3, we discuss the methodology used to estimate the compressibility of individual hydration shells and the results we obtain. We comment on the scope and limitations of the SSM approach in Section 4 and conclude in Section 5 with our main results and future directions.

## 2. Methods

In this work, a generic hydrophobic polymer model, developed by Zangi et al. [30], was used to simulate aqueous polymer systems at various strengths of the polymer-water repulsive interaction. The polymer chain consisted of 40 uncharged Lennard-Jones (LJ) beads ($\sigma_{p}$ = 0.4 nm and $\epsilon_{p}$ = 1.0 kJ mol${}^{-1}$) connected via rigid bonds of length of 0.153 nm. The simulation system was composed of the 40-mer chain, which was periodically replicated in the Z-direction to obtain an infinitely long polymer chain [31], along with 5000 TIP4P/2005 water [32] molecules. The box size along the Z-direction, $l_{z}$, was equal to the end-to-end distance $R_{\mathrm{ee}}$ of the 40-mer chain, which was fixed at 6.11 nm in all simulations to ensure a fully stretched polymer conformation. Thus, unlike in the original model [30], the equilibrium bond angles were kept at 180${}^{\circ }$. Additionally, position restraints, involving a harmonic potential with a force constant of $10^{5}$ kJ mol${}^{-1}$nm${}^{-2}$, were applied on each polymer bead to keep the chain conformation fixed.

The MD simulations were performed using the Gromacs 2019.3 [33] package. All bonds were constrained using the LINCS algorithm [34]. For the van der Waals interaction, a cutoff of 1.4 nm was used without long range dispersion corrections. Long range electrostatic interactions were calculated using the particle mesh Ewald method [35] with a real space cutoff of 1.4 nm, a grid space of 0.12 nm, and an interpolation order of $10^{-4}$. The pressure and temperature were fixed at 1 bar and 300 K, respectively. The simulation systems were energy minimized using the steepest descent method until convergence and subsequently equilibrated in the NVT ensemble for 1 ns using the velocity-rescale thermostat [36]. This was followed by a 1 ns simulation run under NPT conditions using the Nośe-Hoover thermostat [37], with $\tau_{T}$ =1.0 ps, and the Berendsen barostat [38], with $\tau_{P}$ = 2.0 ps. In the NPT simulations, the end-to-end distance of the chain ($R_{\mathrm{ee}}=l_{z}$) was kept constant by applying a semi-isotropic pressure coupling, which allowed box fluctuations only in the X- and Y-directions. Subsequently, a 2 ns NPT run was performed using the Nośe-Hoover thermostat [37] ($\tau_{T}$ = 1.0 ps) and the Parinello–Rahman barostat [39] ($\tau_{P}$ = 2.0 ps). This NPT run was followed by 5 cycles of NPT (using Nośe-Hoover thermostat and Parinello–Rahman barostat) and NVT (using Nośe-Hoover thermostat) runs of 1 ns duration each in order to ensure that the pressure in the NVT simulations fluctuates around 1 bar. The last NVT simulation was extended by 10 ns to generate the production trajectory, which was used for subsequent analysis. For all simulations, an integration time-step of 2 fs was used, and the system snapshots were collected every 1 ps.

**Figure 1:**
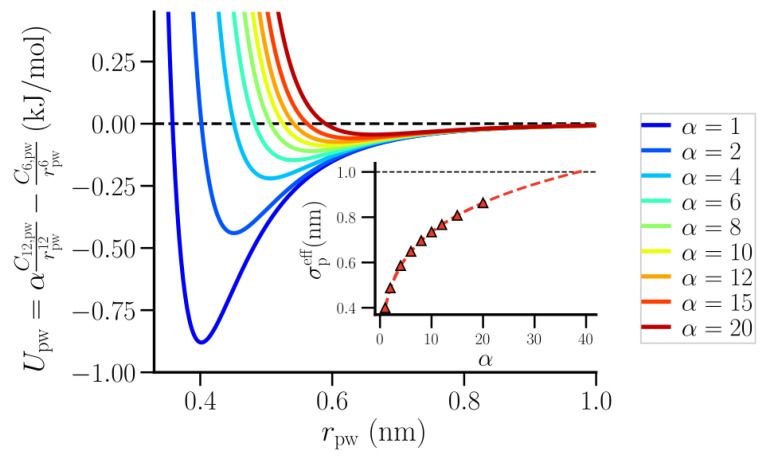
The polymer-water interaction potential, $U_{\mathrm{pw}}$, at various repulsive interaction strength, α. The inset shows the relation between the effective size of the polymer beads, $\sigma_{p}^{\mathrm{eff}}$, and α.

The polymer-water interaction was modeled using an LJ 12-6 potential with the form,

(4) $$U_{\mathrm{pw}}=\alpha \frac{C_{12,\mathrm{pw}}}{r_{\mathrm{pw}}^{12}}-\frac{C_{6,\mathrm{pw}}}{r_{\mathrm{pw}}^{6}},$$

where $C_{12,\mathrm{pw}}(=4\epsilon_{\mathrm{pw}}\sigma_{\mathrm{pw}}^{12}$) and $C_{6,\mathrm{pw}}\left(=4\epsilon_{\mathrm{pw}}\sigma_{\mathrm{pw}}^{6}\right)$ are, respectively, the repulsive and attractive contributions to the potential and α is a scaling factor for the repulsive interaction. The polymer- water interaction parameters, $\epsilon_{\mathrm{pw}}$ and $\sigma_{\mathrm{pw}}$, were calculated using Lorenz–Bertholet mixing rules. The repulsive part of the potential was systematically tuned such that at low repulsion (low α) the polymer resembles an infinite string of overlapping small hydrophobic beads while at high repulsion (high α) it resembles an extended hydrophobic surface. The simulations were performed with α = 1, 2, 4, 6, 8, 10, 12, 15, and 20. As shown in Figure 1, an increase in α led to an increase in the minimal distance of approach between the polymer and water, and a corresponding decrease in their attractive interaction strength. It is, therefore, possible to calculate the effective size of the polymer beads ($\sigma_{p}^{\mathrm{eff}}$) in terms of the interaction parameters and α:

(5) $$\begin{array}{cc} U_{\mathrm{pw}} & =\alpha \frac{C_{12,\mathrm{pw}}}{r_{\mathrm{pw}}^{12}}-\frac{C_{6,\mathrm{pw}}}{r_{\mathrm{pw}}^{6}}=\frac{C_{12,\mathrm{pw}}^{\mathrm{eff}}}{r_{\mathrm{pw}}^{12}}-\frac{C_{6,\mathrm{pw}}^{\mathrm{eff}}}{r_{\mathrm{pw}}^{6}},\\ \sigma_{\mathrm{pw}}^{\mathrm{eff}} & =\alpha^{1/6}\sigma_{\mathrm{pw}},\\ \sigma_{p}^{\mathrm{eff}} & =2\sigma_{\mathrm{pw}}^{\mathrm{eff}}-\sigma_{\mathrm{ww}}. \end{array}$$

The inset in Figure 1 shows the change in $\sigma_{p}^{\mathrm{eff}}$ as a function of α. $\sigma_{p}^{\mathrm{eff}}$ increased with increasing α, approaching a value of 1 nm at α = 40.

A pure water system, which was used as the reference for the analysis of hydration shell properties, consisted of 8000 TIP4P/2005 water molecules in a cubic simulation box. This system was equilibrated following the same protocol that we used for the aqueous polymer system, using repeated NPT-NVT equilibration cycles. This was done to ensure that the density of the system in the NPT ensemble equilibrates and the pressure in the NVT production trajectory fluctuates around 1 bar and well represents the ambient conditions. Unless and otherwise specified, all reference analyses for hydration shell properties were performed using this simulation.

As we are interested in the properties of the first few polymer hydration shells, the small systems were chosen to be cylindrical shells around the linearly extended polymer chain. This way, only the direct effect of polymer-water interaction is included in the analysis while avoiding any effect of polymer conformational fluctuations. There have been previous studies where small systems of various shapes were used to sample thermodynamic properties, which were found to scale identically with the surface to volume ratio of the small systems [15]. For all these shapes (sphere, cube, and polygons), the size of the small system was measured in terms of the diameter or edge length. On the other hand, a cylindrical shape of the small system involves two length parameters, the radius and the height. Therefore, to test our current approach involving cylindrical observation volumes, we first used it to estimate the bulk isothermal compressibility of SPC/E water [40]. The details of the analyses on bulk SPC/E water and aqueous polymer systems are discussed in the next section along with the corresponding results.

In addition to $\Delta 1/\Gamma_{s}^{\infty }$ and $\Delta \chi_{s}$ of the polymer hydration shells, changes in the structural order of the water molecules in the first hydration shell of the hydrophobic polymer were quantified in terms of the tetrahedral order parameter $q_{\mathrm{tet}}$. For a central oxygen atom *i* surrounded by the nearest neighbor oxygen atoms {j,k}, $q_{\mathrm{tet}}$ of the central oxygen was calculated as [41,42]:

(6) $$q_{\mathrm{tet},i}=1-\frac{3}{8}\sum_{j=1}^{3}\sum_{k=j+1}^{4}{\left(\cos\psi_{ijk}+1/3\right)}^{2},$$

where $\psi_{ijk}$ is the angle between the bond vectors **r**${}_{ij}$ and **r**${}_{ik}$. As the water molecules on the outer surface of the first hydration shell can have hydrogen bond neighbors in the second shell, those instances were included in the calculations to compute $q_{\mathrm{tet}}$ for the first shell as a function of α. For comparison, $q_{\mathrm{tet}}$ for shells in pure water were also computed using the polymer hydration shell widths corresponding to various α values.

In all cases, the 10 ns long production trajectory was divided into four windows of 2.5 ns each and the thermodynamic properties were calculated over the windows. The resulting data was averaged to get the mean values of the quantities along with the corresponding standard error.

## 3. Results and Discussion

### 3.1. Isothermal Compressibility of SPC/E Water: Sampling Fluctuations in Cylindrical Observation Volumes

**Figure 2:**
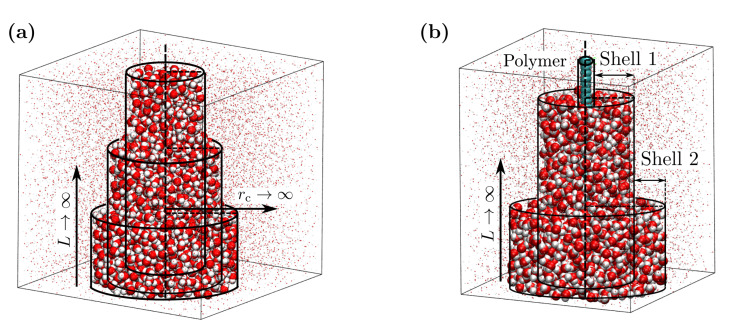
Observation volumes used in this work to estimate thermodynamic properties. (**a**) The isothermal compressibility of SPC/E water is estimated using cylindrical observation volumes, where the TL corresponds to both 1/L→0 and $1/r_{c}\to 0$. (**b**) The thermodynamical quantities pertaining to polymer hydration shells are estimated using concentric cylinders as observation volume. Here, the TL corresponds to 1/L→0.

**Figure 3:**
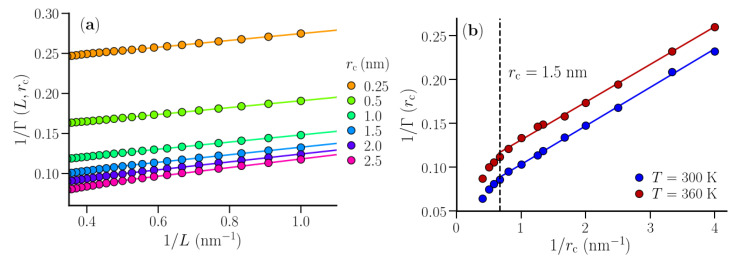
(**a**) $1/\Gamma (L,r_{c})$ profiles for the cylindrical shells with radius $r_{c}$ in SPC/E water at *T* = 300 K, as a function of 1/L. The lines are linear fits to the data in the range *L* = 1 - 2 nm. (**b**) $1/\Gamma (r_{c})$ for the cylindrical shells as a function of $1/r_{c}$ at *T* = 300 K, 360 K. The lines are linear fits to the data in the range $r_{c}<$ 1.5 nm.

A system of 8000 SPC/E water molecules was simulated (following the protocol discussed above) at 300 K and 360 K to benchmark the small box method using cylindrical shapes of the subvolumes. A point close to the center of the simulation box (with box size ∼ 6.23 nm) was identified through which the reference axis was fixed along the Z-direction. Around this reference axis, particle number fluctuations were sampled in cylindrical observation volumes using the oxygen centers of the water molecules (Figure 2a). The radius of the cylinder ($r_{c}$) was varied between 0.25 nm to 2.5 nm. For a fixed value of $r_{c}$, the height of the cylinder (*L*) was varied in steps of 0.1 nm, starting from *L* = 0.1 nm (the smallest subvolume). Here, $r_{c}$ and *L* together represented a small subvolume. For a given $r_{c}$, the observation cylinder was translated along the fixed axis, also in steps of 0.1 nm, for a given value of *L* and fluctuations were sampled. This led to improved statistics, especially for the values of *L*, which are less than half the box size. For a given $r_{c}$, $1/\Gamma (L,r_{c})$ was obtained from the observed particle number fluctuations (Equation (3)). In the resulting data for $1/\Gamma (L,r_{c})$ vs. 1/L (Figure 3a), a linear regime was identified between *L*=1 nm and 2 nm across all values of $r_{c}$. This linear regime was extrapolated to 1/L→0, following Equation (1), to obtain $1/\Gamma (r_{c})$, which characterizes particle number fluctuations in an infinitely long cylinder of radius $r_{c}$ in bulk water. It should be noted that the simulation box behaves like a particle bath for the open small subvolumes. As the size of the subvolume becomes comparable to that of the simulation box, this assumption breaks down. Thus, data for large *L* values suffer from finite size effects and insufficient statistics. On the other hand, for very small values of *L* corresponding to a few particle diameters, $1/\Gamma (L,r_{c})$ deviates from the scaling relation (Equation (1)) as well. Therefore, the data for intermediate values of *L* were considered for estimating the limiting bulk properties.

The data for $1/\Gamma (r_{c})$ vs. $1/r_{c}$ presented a linear regime for $r_{c}<$ 1.5 nm (Figure 3b). This linear regime was extrapolated to obtain $1/\Gamma^{\infty }$, which is the thermodynamic limiting value of 1/Γ corresponding to both 1/L and $1/r_{c}\to 0$. The values of $1/\Gamma^{\infty }$ (at 300 K and 360 K) were then used to estimate the bulk isothermal compressibility of SPC/E water, using Equation (2). We estimated the values of $\chi_{T}$ for SPC/E water to be 43.3$\times 10^{-6}$ bar${}^{-1}$ and 56.0$\times 10^{-6}$ bar${}^{-1}$ at 300 K and 360 K, respectively. These values are comparable to those reported by Heidari et al. [43], who used the finite size correction approach to estimate the compressibility of SPC/E water in the TL using the scaling of particle number fluctuations with system size in cubical subvolumes. The values are also in agreement with those estimated using volume fluctuation methods [44,45,46]: 46.1$\times 10^{-6}$ bar${}^{-1}$ and 57.7$\times 10^{-6}$ bar${}^{-1}$ at 298.15 K and 360 K, respectively. The agreement confirms the validity of the current implementation of the SSM. In principle, one can also calculate the compressibility for each small subvolume $\chi (L,r_{c})$ and use the same 1/L scaling to extrapolate it to the compressibility of the infinite cylindrical shell $\chi (r_{c})$, which in the limit $1/r_{c}\to 0$ will give the bulk isothermal compressibility $\chi_{T}$ [47]. Thus, with the height of the shell L→∞, one can assign $1/\Gamma (r_{c})$ and $\chi (r_{c})$ to be the properties of the particular shell with radius $r_{c}$. This observation asserts the rationale behind choosing cylindrical observation shells to study the thermodynamic properties of the hydration shells of a polymer.

### 3.2. Thermodynamic Properties of Polymer Hydration Shells

Thermodynamic properties for the individual hydration shells of the hydrophobic polymer were estimated from the fluctuations in number of water molecules therein. Unlike the cylindrical observation volumes in the SPC/E water case, the subvolumes were now concentric cylindrical shells around the linearly stretched polymer chain (Figure 2b). The boundaries of these shells were identified based on the local water number density around the polymer. At any value of α, this density was characterized by the proximal radial distribution function (pRDF), $g_{p}\left(r\right)$. Unlike the radial distribution function, g(r), where particles are identified and binned in spherical shells, the particles in this case were binned in cylindrical shells around the polymer.

**Figure 4:**
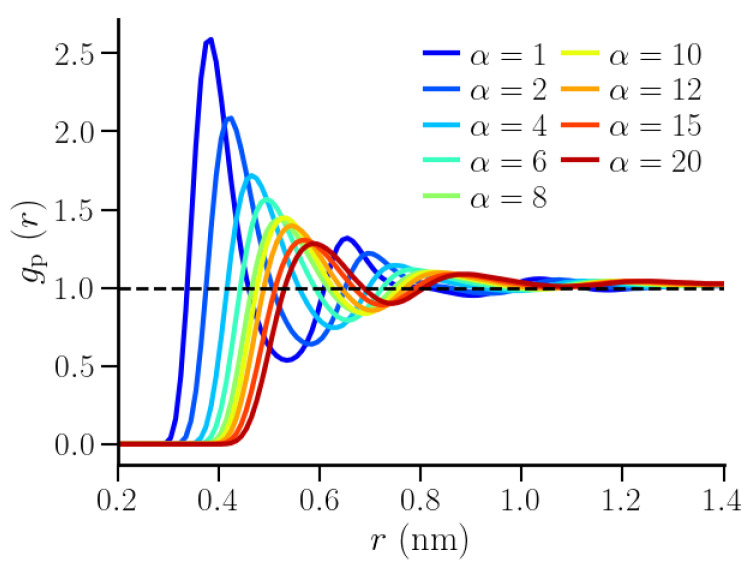
Proximal polymer-water RDFs for various strengths of repulsive interaction parameter α.

Figure 4 shows the polymer-water pRDFs for different values of the repulsive interaction strength, α. As expected, the curves shifted to larger values of *r* while the peak heights decreased, indicating that polymer-water density correlations decreased with increasing values of α. Correspondingly, the shell widths also increased with increasing α (Figure S1 in the Supplementary). Similar to the dependence of the effective polymer bead size, $\sigma_{p}^{\mathrm{eff}}$ (inset of Figure 1), on α, the pRDFs changed significantly upon increasing α between α=1 and α=8, while the changes were smaller for larger values of α. The thermodynamic properties of interest were sampled in the first two hydration shells. The inner boundary of the first hydration shell was identified as the distance $r_{1}$ at which $\rho \int_{0}^{r_{1}}2\pi rl_{z}drg_{p}\left(r\right)>1$. The subsequent minima in the $g_{p}\left(r\right)$ curves were identified as the boundaries of the respective hydration shells.

**Figure 5:**
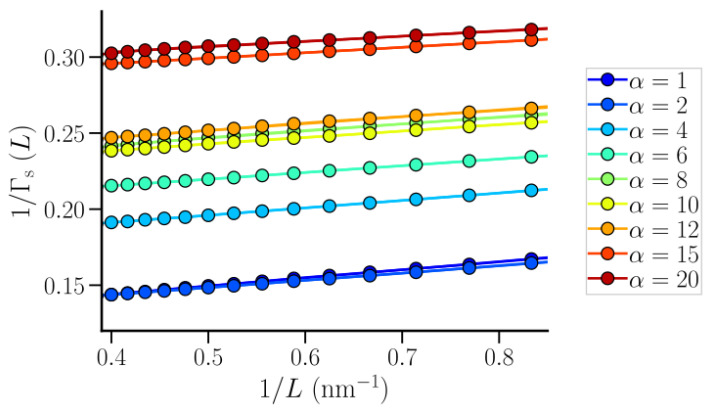
$1/\Gamma_{s}\left(L\right)$ profiles for the first polymer hydration shell for different values of the repulsive interaction parameter α. The lines are linear fits to the data in the range *L* = 1.2–2.5 nm.

The particle number fluctuations were sampled and fitted to calculate the thermodynamic limiting quantities. Only the oxygen centers were used in the calculation. As in the case of SPC/E water, *L* was sampled in steps of 0.1 nm, starting from *L* = 0.1 nm, and the observation volumes were translated along the polymer axis (also in steps of 0.1 nm) for better statistics. Figure 5 shows the data for $1/\Gamma_{s}\left(L\right)$ as a function of 1/L for the first hydration shell of the polymer, over the range of α used in this work. The profiles were found to be linear over the intermediate *L* range, while the curves shifted upward with increasing α indicating enhanced density fluctuations with increasing polymer-water repulsion. The data over the range of *L* between 1.2 nm and 2.5 nm were fitted to a straight line to estimate $1/\Gamma_{s}^{\infty }$, which was subsequently used to estimate $\chi_{s}$. The values of $1/\Gamma_{s}^{\infty }$ and $\chi_{s}$ estimated this way are properties of the hydration shells that are essentially infinite along the polymer axis, but finite in the other two dimensions and thus, do not pertain to a bulk system.

To evaluate the net effect of the polymer, the same properties, here denoted as $1/\Gamma_{s}^{•,\infty }$ and $\chi_{s}^{•}$, were also evaluated in shells of exactly the same widths in a pure TIP4P/2005 water system where the polymer was absent. The inverse thermodynamic correction factor, $1/\Gamma_{s}^{•,\infty }$, of these shells depended on α through the shell widths, which for the first hydration shell, increased with increasing α (Figure S1 in the Supplementary). Both $1/\Gamma_{s}^{•,\infty }$ and $\chi_{s}^{•}$ were found to decrease with increasing α (Figures S2a and S3a in the Supplementary). It should be noted that unlike for bulk water where the TL corresponds to both 1/L and $1/r_{c}\to 0$, the hydration shell specific thermodynamic quantities were estimated in the limit to 1/L→0 alone, and therefore the values of the corresponding thermodynamic quantities are expected to be different. The respective thermodynamic quantities for polymer hydration shells and the corresponding shells in pure water were subtracted to estimate the sole effect of the polymer on the hydration shell thermodynamics for varying α,

(7) $$\begin{array}{cc} \Delta 1/\Gamma_{s}^{\infty } & \equiv 1/\Gamma_{s}^{\infty }-1/\Gamma_{s}^{•,\infty },\\ \Delta \chi_{s} & \equiv \chi_{s}-\chi_{s}^{•}. \end{array}$$

**Figure 6:**
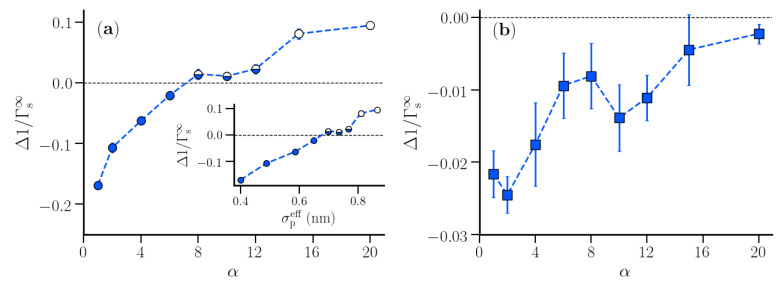
$\Delta 1/\Gamma_{s}^{\infty }$ profiles for the first (**a**) and second (**b**) polymer hydration shell for various strengths α of the repulsive interaction. The inset in (**a**) shows the variation of $\Delta 1/\Gamma_{s}^{\infty }$ as a function of $\sigma_{p}^{\mathrm{eff}}$. In (**a**), the data points are grouped based on their variation with α (see text). The error bars are calculated over four distinct windows in the production trajectory. The lines are guide to the eyes.

Figure 6 presents $\Delta 1/\Gamma_{s}^{\infty }$ for the first two hydration shells of the polymer. $\Delta 1/\Gamma_{s}^{\infty }$ was negative for α=1 and increased with increasing α. A similar increase in density fluctuations was observed in hydration shells of molecular sized hydrophobic solutes [24]. In the first hydration shell, $\Delta 1/\Gamma_{s}^{\infty }$ was negative for α<8, indicating that particle number fluctuations in the first hydration shell were lower than those in shells in pure water. Between α = 8 and 12, the difference between the particle number fluctuations in the first hydration shell and the corresponding shell in pure water almost vanished. For α>8, the particle number fluctuations in the first hydration shell of the polymer were larger than those in the corresponding shell in pure water. The inset in Figure 6 presents $\Delta 1/\Gamma_{s}^{\infty }$ as a function of the effective size of the polymer beads, $\sigma_{p}^{\mathrm{eff}}$. The observed dependence indicated that beyond $\sigma_{p}^{\mathrm{eff}}$ = 0.7 nm, particle number fluctuations in the first hydration shell of the polymer suddenly increased. This observation is similar to the lengthscale crossover observed in the solvation of a hydrophobic cavity in water, when the radius of the cavity is close to 1 nm [23]. The crossover occurs as a result of the disruption of the hydrogen-bonded network of water near the surface of sufficiently large cavities. While water can readily accommodate small solutes without significantly perturbing its hydrogen-bonded network, this is no longer possible near extended surfaces of large hydrophobic solutes. As opposed to a single spherical solute, the hydrophobic polymer in our study resembled a string of small beads at low α-values, while resembling an extended hydrophobic surface when $\sigma_{p}^{\mathrm{eff}}$ approached 0.7 nm (keeping an equilibrium bond length of 0.153 nm).

As opposed to the first hydration shell, the density fluctuations in the second hydration shell were found to be smaller than those in the corresponding pure water shells, even at α = 20 (Figure 6b). An increasing trend in $\Delta 1/\Gamma_{s}^{\infty }$ vs. α, similar to the trend in the first hydration shell, was observed, but with an order of magnitude difference between the two. As water molecules in the second hydration shell are not in direct contact with the polymer surface, the effect of increased polymer-water repulsion is much lower in the second hydration shell as compared to the first hydration shell. This was expected, as the fluctuations were observed to be maximum at the vicinity of the hydrophobic interface [48].

**Figure 7:**
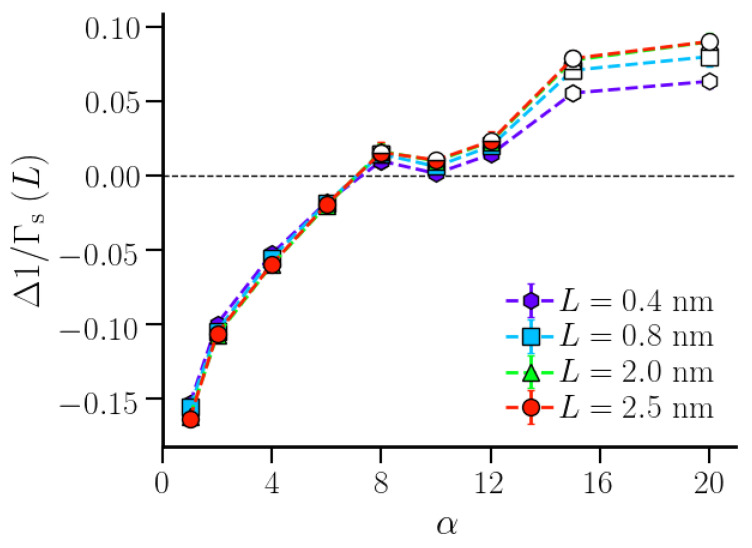
$\Delta 1/\Gamma_{s}\left(L\right)\left(=1/\Gamma_{s}\left(L\right)-1/\Gamma_{s}^{•}\left(L\right)\right)$ profiles for finite-sized first hydration shells with heights *L* = 0.4, 0.8, 2.0, and 2.5 nm as a function of the strength α of the repulsive interaction. The data points are grouped based on their variation with α (as in Figure 6). The error bars are calculated over four distinct windows in the production trajectory. The lines are guide to the eyes.

While we considered the hydration shell to be infinitely long (in the limit 1/L→0), realistic polymer hydration shells are strictly finite. In such a case, it is worthwhile to investigate how strongly $\Delta 1/\Gamma_{s}\left(L\right)$ of a finite hydration shell deviates from that in the macroscopic limit ($\Delta 1/\Gamma_{s}^{\infty }$). We expect that this difference is small for small α-values which correspond to stable hydration shells around small polymer beads with an appreciable bead-water van der Waals attraction. We here consider the variation of $1/\Gamma_{s}\left(L\right)$, $1/\Gamma_{s}^{•}\left(L\right)$, and $\Delta 1/\Gamma_{s}\left(L\right)$ for finite lengths of the first hydration shell corresponding to *L* = 0.4, 0.8, 2.0, and 2.5 nm. While both $1/\Gamma_{s}\left(L\right)$ and $1/\Gamma_{s}^{•}\left(L\right)$ were found to depend on the hydration shell height (Figure S4 in the Supplementary), $\Delta 1/\Gamma_{s}\left(L\right)$ was found to be independent of *L* in the region α≤8, where it was negative (Figure 7). Interestingly, a dependence on the hydration shell dimension was observed for the systems with α>8, corresponding to stronger hydrophobic polymers with larger effective bead sizes and weaker bead-water van der Waals attractions. For these systems, $\Delta 1/\Gamma_{s}\left(L\right)$ increased with *L* (up to *L* = 2.0 nm), indicating that an extended hydrophobic surface created by connected polymer beads caused enhanced water density fluctuations in the first hydration shell.

**Figure 8:**
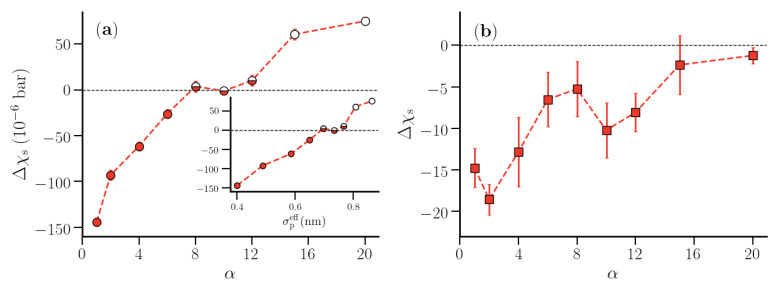
$\Delta \chi_{s}$ profiles for the first (**a**) and second (**b**) polymer hydration shell for various values of the repulsive interaction parameter α. The inset in (**a**) shows $\Delta \chi_{s}$ as a function of $\sigma_{p}^{\mathrm{eff}}$. In (**a**), the data points are grouped based on their variation with α (see text). The error bars are calculated over four distinct windows in the production trajectory. The lines are guide to the eyes.

**Figure 9:**
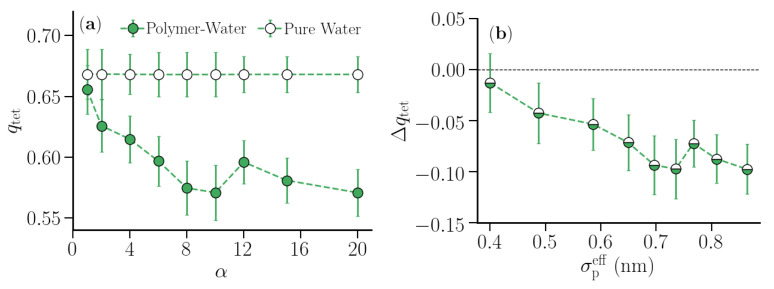
(**a**) Tetrahedral order parameter $q_{\mathrm{tet}}$ for the first polymer hydration shell and the corresponding shell in pure water for various strengths α of the repulsive interaction. (**b**) $\Delta q_{\mathrm{tet}}\;=\;q_{\mathrm{tet}}$(polymer shell) −$q_{\mathrm{tet}}$(water shell) as a function of $\sigma_{p}^{\mathrm{eff}}$. The error bars are calculated over four distinct windows in the production trajectory. The lines are guide to the eyes.

Figure 8 shows the data for $\Delta \chi_{s}$ vs. α for the first and second hydration shell. The compressibility of both hydration shells increased with increasing α. As in the case of $\Delta 1/\Gamma_{s}^{\infty }$, the change in $\Delta \chi_{s}$ for the second hydration shell was rather small. For the first polymer hydration shell, the compressibility was smaller than that for the corresponding shells in pure water until α = 8. Between α = 8 and 12, the hydration shell compressibility was almost identical to the compressibility of the corresponding shells in pure water. For larger α-values there was a noticeable increase in the hydration shell compressibility. This is similar to the case of hydration water of hydrophobic solutes [24], where the local compressibility was found to increase for sufficiently large solutes. Figure 9 shows the tetrahedral order parameter $q_{\mathrm{tet}}$ of the first hydration shell of the polymer corresponding to various values of α and those calculated for shells in pure water. For shells in pure water, $q_{\mathrm{tet}}$ remained almost constant around 0.66, which corresponds to the optimal hydrogen bonding capacity for TIP4P/2005 water [49]. In contrast, the tetrahedral order in the polymer hydration shell decreased by 15% upon increasing α up to α=8, at which point $\Delta \chi_{s}$ became positive. The number of hydrogen bonds per water molecule in the first hydration shell showed a similar dependence (Figure S5).

The increase in density fluctuations, decrease in tetrahedral order, and increase in compressibility in the first hydration shell of the hydrophobic polymer are in line with the trends expected in the case of solvation of hydrophobic solutes in water [23,24,29,50].

## 4. Scope and Limitations

The polymer analyzed in this work is a generic homo-polymer in a restrained, stretched conformation, where the monomeric units are represented by simple beads without any side chains. It is essentially the simplest possible polymer system that could be analyzed in the current framework. Applying the current approach to study chemically realistic polymer systems requires that several technical challenges are addressed. Hill’s formalism [12,13] is framed for a large ensemble of equivalent, independent, and distinguishable small systems. In the present context, this implies that the small system must be at least the size of a monomeric unit and, therefore, *L* has to be chosen as an integer multiple of the monomer length. Therefore, to effectively sample the density fluctuations, very long chemically realistic polymer chains (much longer than the one considered here) need to be considered in the simulations. For the generic polymer used in this work, 1/Γ is insensitive to whether density fluctuations within the small system *L* are sampled by shifting it along the polymer backbone in steps of 0.1 nm or in steps that correspond to a monomeric unit, essentially due to its linearly stretched configuration. The same will not hold for flexible polymers as the monomeric units themselves will differ in terms of the volume they occupy and, therefore, static observation volumes can no longer be used to sample the fluctuations. Therefore, additional methodological progress requires implementation of dynamical observation volumes.

In addition to the application of the SSM to a generic polymer model in pure water, mixed solvents may be considered to be well. In this context, the SSM can be used to provide information on excess chemical potentials and partial molar enthalpies and entropies of solvent components in the solvation shell of the polymer based on analyses of energy and particle number fluctuations and the calculation of KBIs. In particular, in systems where cosolvents and small organic molecules preferentially bind to the polymer, a full characterization of solvation shell properties is needed to address open questions related to the corresponding changes in aqueous polymer solubility [51,52,53]. The SSM provides a route to calculate these properties.

## 5. Conclusions

We have extended the SSM to study the hydration shell compressibility of a generic hydrophobic polymer in water. We identified the hydration shells based on the proximal distribution of water molecules around a linearly extended polymer chain. Water density fluctuations were sampled in small concentric cylindrical shells around the chain, which in the limit of the shell height L→∞ resulted in thermodynamic quantities that could be assigned to the hydration shells. We systematically varied the range of the polymer-water repulsion and observed a crossover behavior in the excess inverse thermodynamic correction factor ($\Delta 1/\Gamma_{s}^{\infty }$) and the excess compressibility ($\Delta \chi_{s}$) of the hydration shells, defined relative to the properties of the same shells in pure water (without polymer). The negative-to-positive crossover observed in $\Delta 1/\Gamma_{s}^{\infty }$ and in $\Delta \chi_{s}$ happened at an intermediate range of the polymer-water repulsion, where the effective polymer bead diameter was around 0.7 nm. We also observed a complementary trend in the tetrahedral order parameter of the shell, which measured the deviation in the hydrogen bonding coordination of water molecules from the ideal tetrahedrally hydrogen-bonded structure.

The observations made in this work were in line with those reported for solvation of spherical hydrophobic solutes in water [23,24,50]. It is well established that small hydrophobic cavities/ solutes are solvated by restructuring of water hydrogen bonds around them, while solvation of large hydrophobic cavities occurs via breakage of water hydrogen bonds near the solute surface. The signatures of these two distinct solvation mechanisms were observed in the present work in the excess polymer hydration shell compressibility.

## Supplementary Materials

The following are available online at https://www.mdpi.com/2079-4991/10/8/1460/s1, Figure S1: Width of polymer hydration shells vs. α, Figure S2: $1/\Gamma_{s}^{\infty }$ and $1/\Gamma_{s}^{•,\infty }$ vs. α, Figure S3: $\chi_{s}$ and $\chi_{s}^{•}$ vs. α, Figure S4: $1/\Gamma_{s}\left(L\right)$ and $1/\Gamma_{s}^{•}\left(L\right)$ vs. α, Figure S5:$\Delta n_{H-\mathrm{bonds}}=n_{H-\mathrm{bonds}}-n_{H-\mathrm{bonds}}^{•}$ vs. α.

## Abbreviations

The following abbreviations are used in this manuscript:

TL	Thermodynamic Limit
SSM	Small-System Method
KBI	Kirkwood-Buff Integral
RDF	Radial Distribution Function
pRDF	Proximal pair correlation function
