# Supplementary material for: Characterizing Polymer Hydration Shell Compressibilities with the Small-System Method
Source: Nanomaterials (Basel). 2020 Jul 25;10(8):1460. doi: 10.3390/nano10081460 (PMC7466400; doi:10.3390/nano10081460)
Supplement: Supplementary file 1 [file nanomaterials-10-01460-s001.pdf]

SUPPORTING INFORMATION

Characterizing Polymer Hydration Shell Compressibilities With The Small System Method

Madhusmita Tripathy,[1, a)] Swaminath Bharadwaj,[1, b)] Shadrack Jabes B.,[1, c)] and Nico F. A. van der Vegt[1, d)]

Eduard-Zintl-Institut für Anorganische und Physikalische Chemie, Technische Universität Darmstadt, 64287 Darmstadt, Germany

[a)]Electronic mail: tripathy@cpc.tu-darmstadt.de

[b)]Electronic mail: bharadwaj@cpc.tu-darmstadt.de

[c)]Electronic mail: barnabas@cpc.tu-darmstadt.de

[d)]Electronic mail: vandervegt@cpc.tu-darmstadt.de

I. $1/\Gamma^{\infty}$ AND χ FOR HYDRATION SHELLS

The width of the first hydration shell was found to increase with increasing α, while that of the second one decreased (Fig. S1). The quantities, $1/\Gamma$ and χ for the polymer hydration shells and for the shells in pure water (without polymer) were calculated using these shell widths as a function of α.

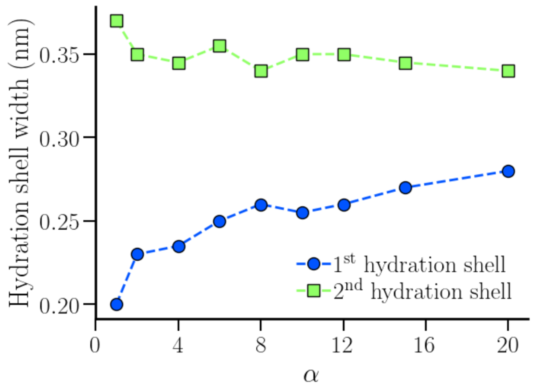

FIG. S1. Width of 1st and 2nd hydration shells for different values of the repulsive strength parameter α.

As the first hydration shell width increased with increasing α, there was a corresponding decrease in density fluctuations in the bulk water shells and a corresponding decrease in the inverse thermodynamic factor, $1/\Gamma_{\mathrm{s}}^{\bullet,\infty}$. On the other hand, with decreasing shell widths, $1/\Gamma_{\mathrm{s}}^{\bullet,\infty}$ of the second shell increased (Fig. S2). For both the shells, $1/\Gamma_{\mathrm{s}}^{\bullet,\infty}$ was found to slowly approach an asymptotic value in the limit of shell width $\to \infty$. Similar trends were observed for χ_{s} (Fig. S3).

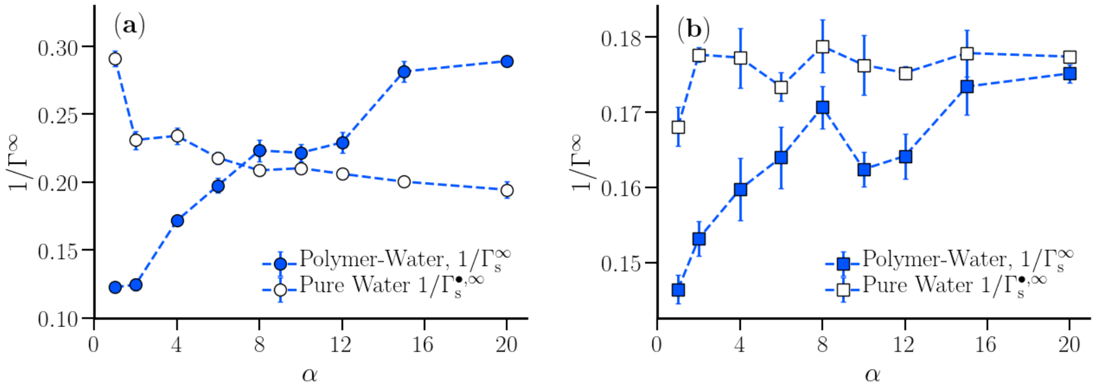

FIG. S2. Profiles of thermodynamic correction factor, $1/\Gamma^{\infty}$ in (a) the 1st hydration shell and (b) the 2nd hydration shell in polymer-water and pure water systems for various values of the repulsive strength parameter α.

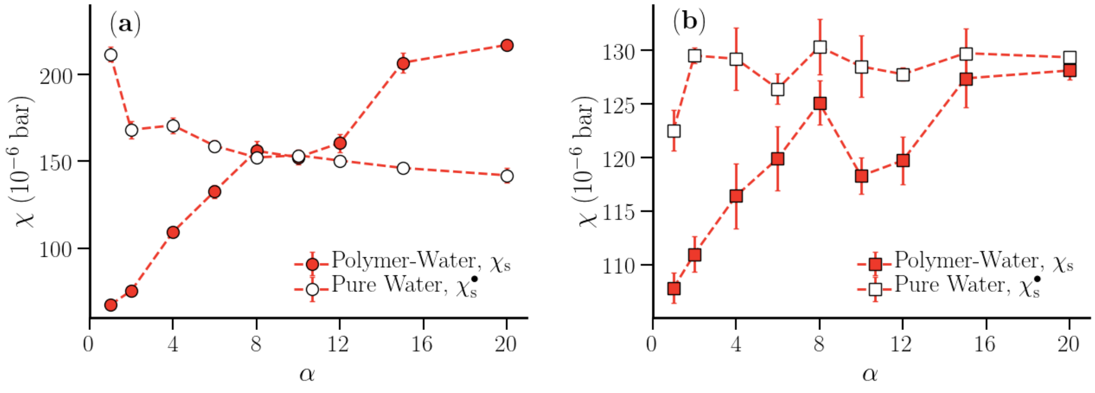

FIG. S3. Profiles of compressibility, χ in (a) the 1st hydration shell and (b) the 2nd hydration shell in polymer-water and pure water systems for various values of the repulsive strength parameter α.

II. DEPENDENCE OF $1/\Gamma$ (L) ON THE SMALL SYSTEM LENGTH SCALE L

$1/\Gamma_s$ and $1/\Gamma_s^{\bullet}$ for finite sized first hydration shells with heights $L = 0.4$, 0.8, 2.0, and 2.5 nm, were found to strongly depend on the hydration shell height (Fig. S4). However, the difference between the profiles reduced as L increased. As shown in the main text (Fig. 7), $\Delta 1/\Gamma_s \equiv 1/\Gamma_s - 1/\Gamma_s^{\bullet}$ was found to be comparable for all values of L considered for $\alpha < 8$.

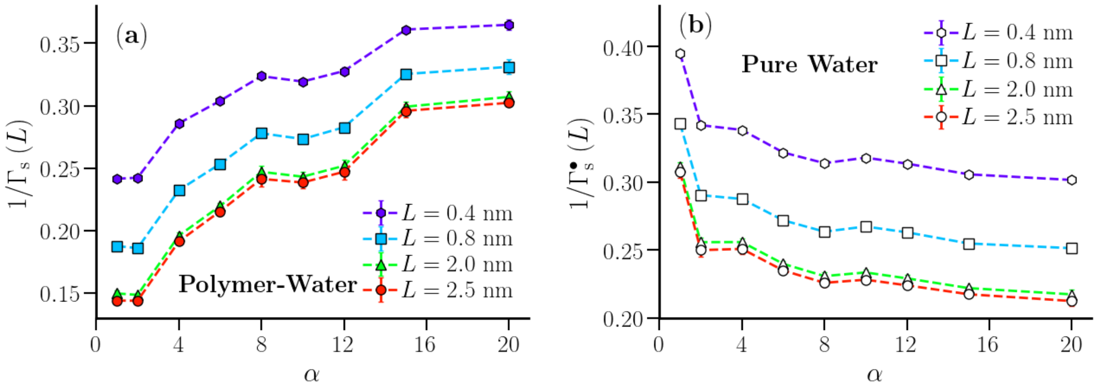

FIG. S4. Profiles of (a) $1/\Gamma_s(L)$ (Polymer-water system) and (b) $1/\Gamma_s^\bullet(L)$ (Pure water) for different small system sizes ($L = 0.4$ nm, 0.8 nm, 2.0 nm, 2.5 nm) for various values of the repulsive strength parameter α.

III. HYDROGEN BONDS

Hydrogen bonds for water molecules within the hydration shell were calculated using the standard criterion implemented in GROMACS. Two water molecules whose oxygen atoms are within a distance of 0.35 nm from one other with an angle of 30° between the O-O and one of the donor O-H bond axes[1] were considered to be hydrogen bonded. Using this definition we computed the average number of hydrogen bonds per hydration water molecule.

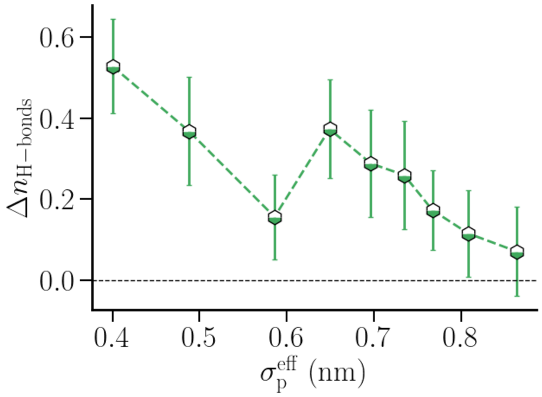

FIG. S5. Profiles of $\Delta n_{\mathrm{H-bonds}} \equiv n_{\mathrm{H-bonds}} - n^\bullet_{\mathrm{H-bonds}}$ for different effective polymer bead sizes.

REFERENCES

[1]A. Luzar and D. Chandler, J. Chem. Phys. **98**, 8160 (1993).
